# Application of Environmental DNA for Monitoring Red Sea Bream Iridovirus at a Fish Farm

**DOI:** 10.1128/Spectrum.00796-21

**Published:** 2021-10-27

**Authors:** Yasuhiko Kawato, Tohru Mekata, Mari Inada, Takafumi Ito

**Affiliations:** a Pathology Division, Nansei Field Station, Fisheries Technology Institute, Japan Fisheries Research and Education Agency, Mie, Japan; Oklahoma State University, College of Veterinary Medicine

**Keywords:** environmental DNA, eDNA, red sea bream iridovirus, red sea bream iridoviral disease, *Megalocytivirus*, iron flocculation, *Pagrus major*, RSIV, RSIVD

## Abstract

Red sea bream iridoviral disease (RSIVD) causes high economic damage in mariculture in Asian countries. However, there is little information on the source of infection and viral dynamics in fish farms. In the present study, the dynamics of RSIV in a fish farm that mainly reared juveniles and broodstocks of red sea bream (Pagrus major) were monitored over 3 years (2016 to 2018) by targeting environmental DNA (eDNA) of seawater. Our monitoring demonstrated that red sea bream iridovirus (RSIV) was detected from the eDNA at least 5 days before an RSIVD outbreak in the juveniles. The viral loads of eDNA during the outbreak were highly associated with the numbers for daily mortality, and they reached a peak of 10^6^ copies/liter seawater in late July in 2017, when daily mortality exceeded 20,000 fish. In contrast, neither clinical signs nor mortality was observed in the broodstocks during the monitoring periods, whereas the broodstocks were confirmed to be virus carriers by an inspection in October 2017. Interestingly, the viral load of eDNA in the broodstock net pens (10^5^ copies/liter seawater) was higher than that in the juvenile net pens (10^4^ copies/liter seawater) just before the RSIVD outbreak in late June 2017. After elimination of all RSIV-infected surviving juveniles and 90% of broodstocks, few RSIV copies were detected in the eDNA in the fish farm from April 2018 onward (fewer than 10^2^ copies/liter seawater). These results imply that the virus shed from the asymptomatically RSIV-infected broodstock was transmitted horizontally to the juveniles and caused further RSIVD outbreaks in the fish farm.

**IMPORTANCE** Environmental DNA (eDNA) could be applied in monitoring waterborne viruses of aquatic animals. However, there are few data for practical application of eDNA in fish farms for the control of disease outbreaks. The results of our field research over 3 years targeting eDNA in a red sea bream (Pagrus major) fish farm implied that red sea bream iridoviral disease (RSIVD) outbreaks in juveniles originated from virus shedding from asymptomatically virus-infected broodstocks. Our work identifies an infection source of RSIVD in a fish farm via eDNA monitoring, and it could be applied as a tool for application in aquaculture to control fish diseases.

## INTRODUCTION

Environmental DNA (eDNA) generally refers to nucleic acids extracted from environmental samples (water, soil, or feces, etc.). eDNA has been studied extensively since the 2000s with numerous applications, such as metagenomics, species detection, or biomass estimation (1, 2). Aquatic animal pathogens are basically waterborne; therefore, eDNA has been studied as a potential tool for monitoring aquatic animal diseases in aquaculture (3–5) and border control (6, 7). Although the term “eDNA” has not been used in fish virus research, eDNA could be applied in monitoring waterborne viruses in combination with virus concentration techniques. As waterborne viruses are generally less abundant in environmental water, several virus concentration techniques have been applied previously, such as ultrafiltration (8, 9), ultracentrifugation (10), polyethylene glycol precipitation (11), the cation-coated filter method (12–14), the iron flocculation method (15–17), and the use of electronegative or -positive filters (18).

Red sea bream iridoviral disease (RSIVD) was first discovered in 1990, following mass mortality of cultured red sea bream (Pagrus major) in Japan. The etiological agent was named red sea bream iridovirus (RSIV) (19). Affected fish exhibit anemia in the gills and an enlarged spleen, in which numerous enlarged cells replicating the virus have been observed (20). Similar viral infections in cultured fish have been reported in several Asian countries, such as infectious spleen and kidney necrosis virus (ISKNV) reported in mandarin fish (Siniperca chuatsi) in China (21, 22), rock bream iridovirus reported in barred knifejaw (Oplegnathus fasciatus) cultures in Korea (23, 24), and turbot reddish body iridovirus (TRBIV) reported in turbot (Scophthalmus maximus) in China (25, 26).

These causative viruses are classified into the genus *Megalocytivirus*, within the family *Iridoviridae* (27). *Infectious spleen and kidney necrosis virus* (ISKNV) is the type species of *Megalocytivirus*, and *Scale drop disease virus* (SDDV) has been recently registered as a new species within the genus by the International Committee on Taxonomy of Viruses (ICTV) (https://talk.ictvonline.org/ictv-reports/ictv_online_report/dsdna-viruses/w/iridoviridae). These viruses have a double-stranded DNA genome approximately 110 kbp in length and an icosahedral virion capsid with a diameter of 140 to 200 nm (20, 27, 28). Based on phylogenetic analysis of the major capsid protein (MCP) gene, the ISKNV species can be divided into three genotypes: the RSIV genotype, reported in marine fish; the ISKNV genotype, reported in both marine and freshwater fish; and the TRBIV genotype, reported mainly in marine flatfish (28–30). RSIVD, caused by the RSIV and ISKNV genotypes, is listed by the World Organization for Animal Health (OIE) because it could have a significant impact on more than 30 fish species in the aquaculture industry if the virus is brought into RSIVD-free countries (31, 32). The distribution of the virus appears to be spreading, as outbreaks of RSIVD have been documented in the United States and the Caribbean Sea (33, 34).

Generally, it is difficult to eliminate the disease from an area where the virus is established; hence, outbreaks of RSIVD often occur in the summer season in Japan. To counter the routine outbreaks of RSIVD in Japan, a formalin-inactivated vaccine has been commercially available since 1999 (35, 36). However, vaccination is sometimes not utilized when the vaccine cost is not acceptable compared to the market value of cultured fish. Although some RSIVD-resistant strains of red sea bream have been developed by marker-assisted selection combined with DNA-based family selection (37), implementing this across all susceptible species for aquaculture will take a long time.

In addition to vaccines and disease-resistant fish strains, activities to identify the infection route and source are important procedures to prevent an epidemic. For RSIVD, horizontal transmission via environmental water is considered to be the main route of infection (20, 38). Regarding the infection sources, fish surviving RSIVD (39, 40), latently infected fish in which viral replication is suppressed at lower temperatures (41, 42), wild fish (43), and bivalves near fish farms (44) are suspected to be the virus carriers or vectors. In Japanese mariculture, since fish of several ages are often reared on the same fish farm, fish surviving RSIVD could be virus carriers and an infection source for newly introduced juveniles in the following year. However, no field study has tested this hypothesis at fish farms.

In the present study, we monitored virus presence in seawater using eDNA over 3 years (2016 to 2018) in a Japanese fish farm where RSIVD occurred in juvenile red sea bream. The eDNA, including viruses in the seawater, was concentrated using the iron flocculation method, followed by DNA extraction (16). The viral loads of the eDNA were quantitatively monitored using real-time PCR. The application of eDNA for monitoring RSIV and the infection source for the RSIVD outbreak in the fish farm are discussed based on the results of the investigation.

## RESULTS

### RSIV detection in red sea bream juveniles and broodstock.

**FIG 1:**
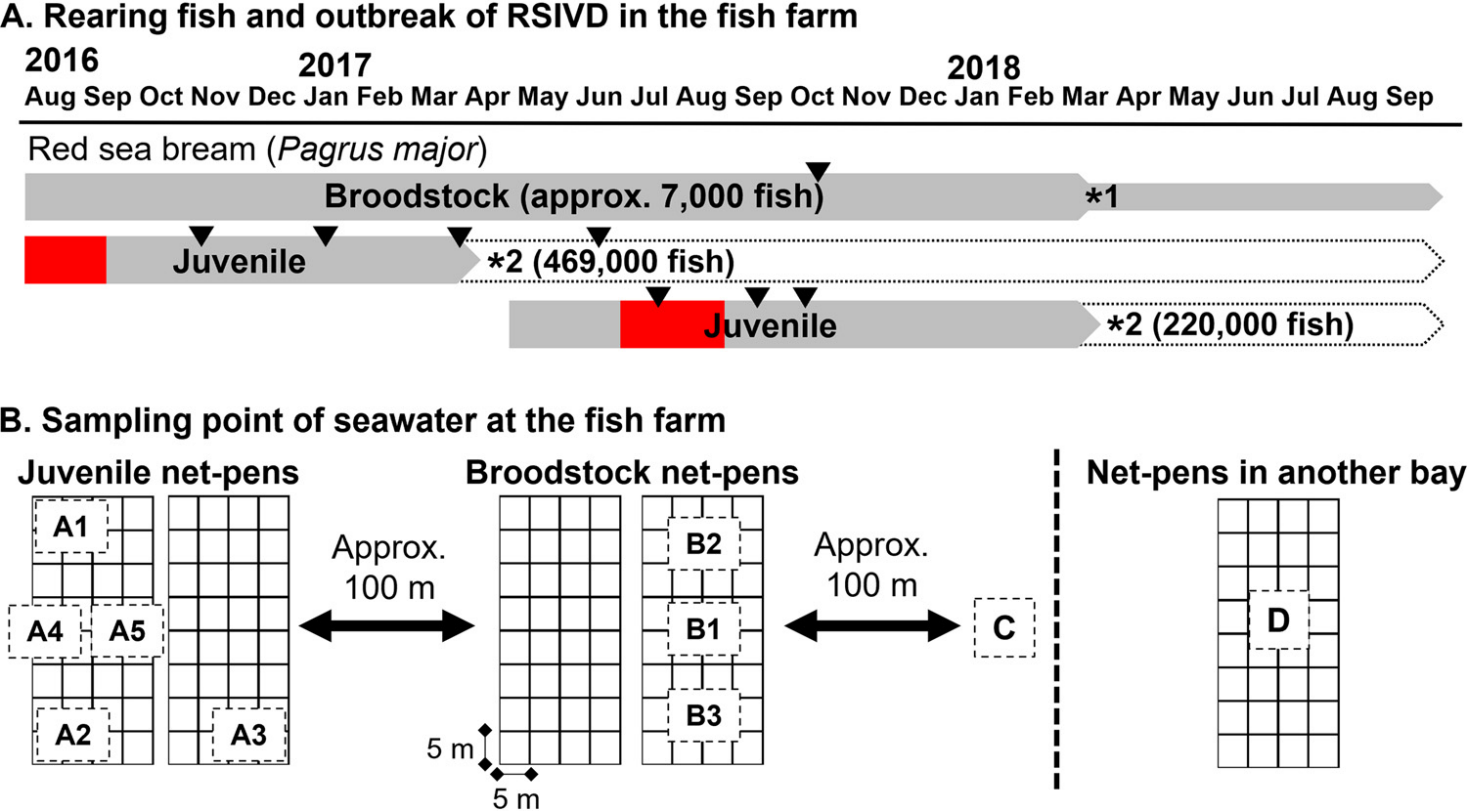
Overview of this study. (A) Gray-filled boxes and red-filled boxes indicate the rearing fish and RSIVD outbreaks, respectively, in sampling points A and B at the fish farm. Arrowheads indicate fish samples used for real-time PCR. *1, 90% of the broodstock were sold; *2, all rearing fish were transferred to new net pens in another bay area (sampling point D) or sold. (B) A1 to A5, net pens for red sea bream juveniles; B1 to B3, net pens for red sea bream broodstock; C, outside the fish farm; D, net pens in another bay area.

**FIG 2:**
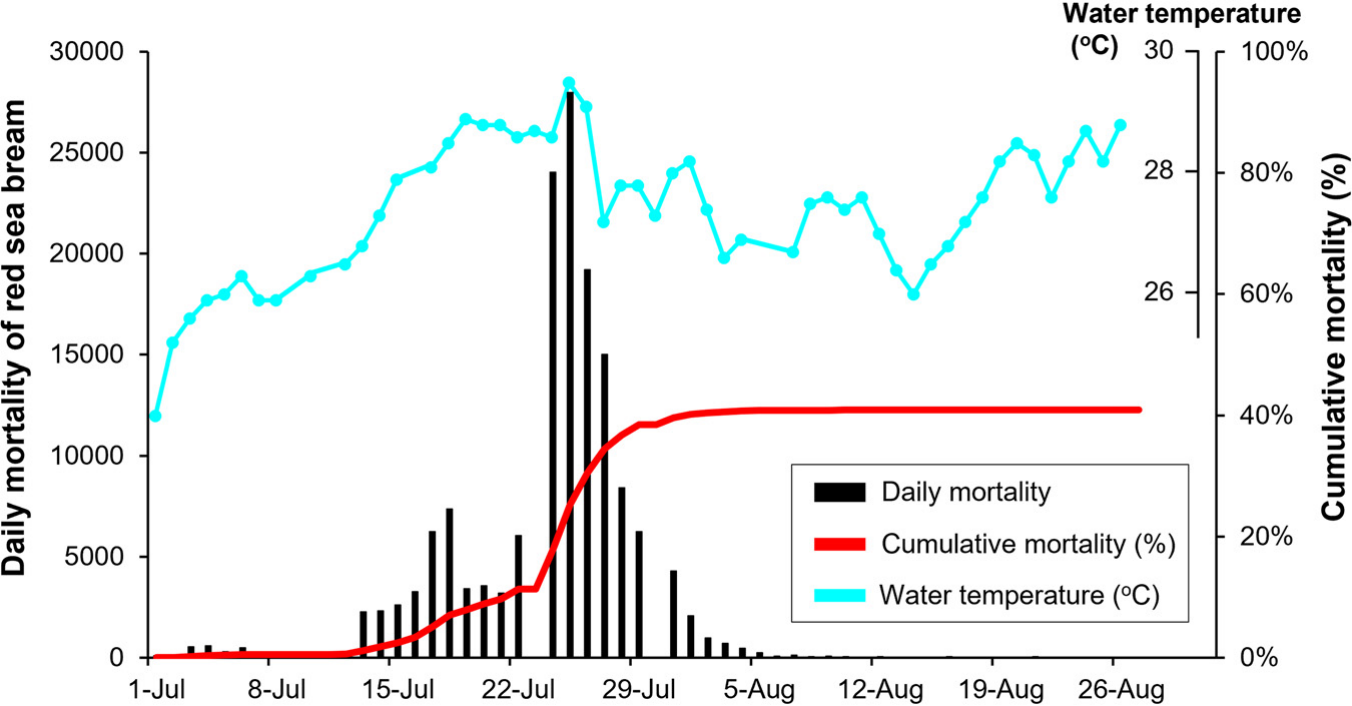
Mortality record of red sea bream juveniles in July and August 2017. In total, 372,000 red sea bream juveniles had been reared in the fish farm since May 2017, and the first mortality due to RSIVD was recorded on June 27.

An overview of fish sampling is shown in Fig. 1A The results of RSIV detection in the red sea bream juveniles and broodstock are shown in Tables 1 and 2, respectively. Since the RSIV genome was frequently detected in the spleen and kidney samples from November 2016, the spleen was used for further investigation in surviving fish. The mean frequency of RSIV detection in surviving fish in 2016 was 72.5% ± 12.9%. However, viral loads were low (10^2.4 ± 0.6^ copies/mg tissue) in the surviving fish, and the detection rate of RSIV in the surviving fish did not decrease even after 10 months of outbreak. For the outbreak in 2017 (Fig. 2), high levels (10^8.0 ± 0.4^ copies/mg tissue) of viral DNA were detected in fish that died during the peak of the outbreak (July 27). In contrast, the viral loads decreased in the surviving fish in September and October 2017 as the outbreak ceased. Although no RSIVD occurred in the broodstock, the RSIV genome was detected in all spleen and kidney samples obtained in October 2017. The observation that RSIV was detected in the pyloric caeca and intestine tissues with viral loads similar to those of the spleen and kidney tissues was a novel finding (Table 2).

**TABLE 1:** Results of RSIV real-time PCR for the surviving and dead red sea bream juveniles

Sampled fish, sampling date^*a*^	No. of fish sampled	BW (g)	Dead/surviving	Organ	No. positive for RSIV/no. tested	No. of RSIV genomes/mg tissue^*b*^
Surviving fish						
7 Nov 2016	5	35.4	Surviving	Fin	0/5	Und
				Gill	0/5	Und
				Intestine	1/5	10^2.1^
				Liver	0/5	Und
				Kidney	3/5	10^2.1 ± 1.0^
				Spleen	3/5	10^2.5 ± 0.7^
10 Jan 2017	15	98.7	Surviving	Spleen	11/15	10^2.2 ± 0.4^
12 Apr 2017	15	129.4	Surviving	Spleen	10/15	10^2.9 ± 0.4^
8 Jun 2017	10	129.5	Surviving	Spleen	9/10	10^1.8 ± 0.9^

Introduced juveniles						
27 Jul 2017	5	15.2	Dead	Spleen	5/5	10^8.0 ± 0.4^
21 Sep 2017	5	55.3	Surviving	Spleen	5/5	10^3.0 ± 0.5^
6 Oct 2017	5	48.7	Surviving	Spleen	5/5	10^3.3 ± 0.2^

a Surviving fish after the outbreak in August 2016 and red sea bream juveniles that were newly introduced in May 2017 were sampled.

b Und, all examined samples were under the detection limit (10^1^ copies/mg tissue).

**TABLE 2:** RSIV genome numbers in the tissue samples of red sea bream broodstock

Organ	No. of RSIV genomes in the organ (copies/mg) in fish no.^*a*^:		
	1	2	3
Spleen	9.6E+01	6.7E+02	3.8E+03
Kidney	2.4E+02	1.5E+02	7.7E+02
Liver	Und	Und	Und
Heart	Und	Und	5.1E+01
Stomach	2.6E+01	Und	Und
Pyloric caeca	2.3E+01	2.5E+03	Und
Intestine	Und	3.8E+02	1.4E+03
Brain	Und	Und	2.5E+01
Caudal fin	2.9E+01	Und	1.1E+02
Gill	Und	Und	Und
Gonad	Und	4.2E+01	Und

a Und, under the detection limit (1.0E+01 copies/mg tissue).

### RSIV dynamics based on eDNA.

**FIG 3:**
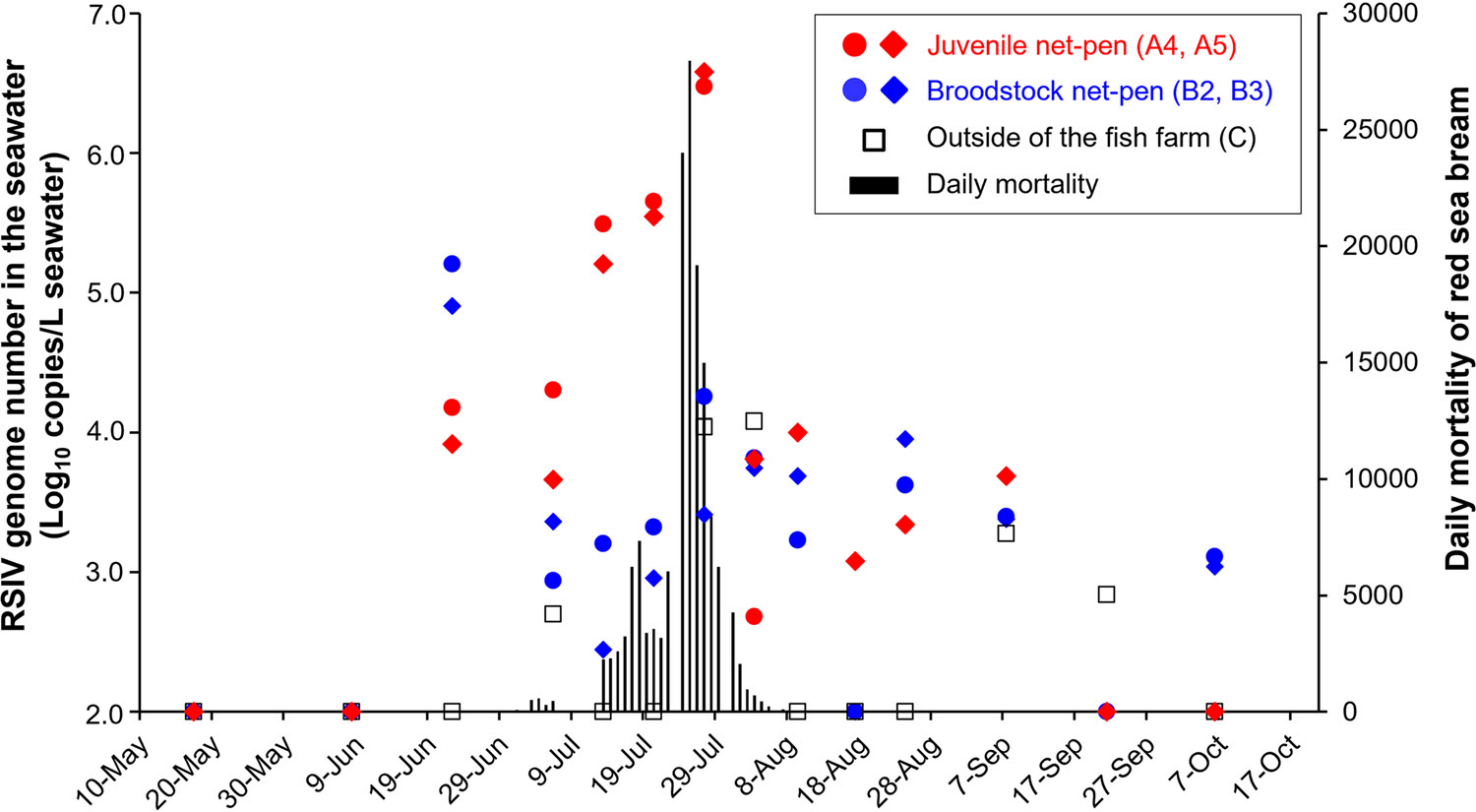
Relationship between viral loads in the seawater and daily mortality of red sea bream juveniles on the fish farm in 2017.

The sampling points for eDNA derived from seawater are shown in Fig. 1B. The RSIV genome was detected in the eDNA of the fish farm in October 2016, even once the RSIVD outbreak had ceased. The viral load of the broodstock net pen was 10 times higher than those of the juvenile net pens where surviving fish were reared (Table 3). In contrast, no RSIV was detected from the seawater derived from sampling point C, which was approximately 100 m from the broodstock net pen. The shedding of RSIV became almost undetectable when the water temperature was lower than 20°C (Table 3). No RSIV genomes were detected in the seawater of the fish farm between February and May 2017, but RSIV was suddenly detected on June 22 in the broodstock and new juvenile net pens at viral loads of 10^5^ and 10^4^ copies/liter seawater, respectively (Table 4), despite the removal of the surviving fish from the 2016 RSIVD outbreak in the cultured area. As daily mortality due to RSIVD rose at the end of June 2017, the viral loads in the juvenile net pens increased along with increasing daily mortality and reached a peak on July 27 at 10^6^ copies/liter seawater (Table 4 and Fig. 3). Then, the viral loads decreased as mortality ceased, and RSIV was not detected in the juvenile net pen from September 21 onwards (Table 4). For the broodstock net pens, the viral load peaked on June 22. Then, it remained at approximately 10^3^ copies/liter seawater until October 2017, indicating that the viral shedding of the apparently healthy broodstock was more prolonged than that of the surviving fish after the RSIVD outbreak. Sampling point D, to which the surviving fish in 2016 were transferred, was monitored to investigate whether the fish surviving RSIVD shed the virus into the seawater environment. In fact, RSIV was detected at sampling point D, but no mortality due to RSIVD was recorded in the surviving fish, and the viral loads and detection frequency were low (Table 4). Almost no RSIV genomes were detected in the seawater of the fish farm in 2018 and 2019 (Table S1 in the supplemental material).

**TABLE 3:** Viral loads in the environmental seawater between October 2016 and April 2017

Yr	Date	Water temp (°C)	RSIV genome no. (copies/liter seawater) at sampling point^*a*^:				
			A1	A2	A3	B1	C
2016	18 Oct	24	1.5E+02	1.5E+02	5.6E+02	4.2E+03	–
	25 Oct	23	4.7E+02	6.6E+02	2.7E+02	1.6E+04	Und
	7 Nov	21	2.6E+02	Und	Und	Und	Und
2017	10 Jan	14.4	Und	Und	Und	1.2E+02	Und
	9 Feb	12.5	Und	Und	Und	Und	Und
	21 Mar	12.7	Und	Und	Und	Und	Und
	12 Apr	15.3	Und	Und	Und	Und	Und

a A1, A2, and A3, net pens for red sea bream juveniles; B1, net pens for red sea bream broodstock; C, sampling point outside the fish farm; –, no data; Und, under the detection limit (1.0E+02 copies/liter seawater).

**TABLE 4:** Viral loads in the environmental seawater after the introduction of new juveniles in May 2017

Yr	Date	Water temp (°C)	No. of RSIV genomes (copies/liter seawater) at sampling point^*a*^:					
			A4	A5	B2	B3	C	D
2017	17 May	19.4	Und	–	Und	Und	Und	–
	8 Jun	21	Und	–	Und	Und	Und	1.9E+03
	22 Jun	22.6	8.3E+03	1.5E+04	1.6E+05	8.0E+04	Und	–
	6 Jul	26.5	4.6E+03	2.0E+04	8.7E+02	2.3E+03	5.0E+02	Und
	13 Jul	26.8	2.8E+05	5.6E+05	1.6E+03	2.0E+03	1.6E+03	Und
	20 Jul	28.8	6.3E+05	8.5E+05	2.1E+03	4.5E+03	2.8E+03	3.8E+02
	27 Jul	27.3	3.8E+06	3.0E+06	1.8E+04	2.6E+03	1.1E+04	Und
	3 Aug	26.6	6.5E+03	4.8E+02	6.6E+03	5.6E+03	1.2E+04	Und
	9 Aug	27.6	1.0E+04	–	1.7E+03	4.9E+03	Und	8.0E+02
	17 Aug	27.2	1.2E+03	–	Und	Und	Und	Und
	24 Aug	28.7	2.2E+03	–	4.2E+03	9.0E+03	Und	Und
	7 Sep	25.9	4.9E+03	–	2.5E+03	2.4E+03	1.9E+03	Und
	21 Sep	24.8	Und	–	Und	Und	6.9E+02	Und
	6 Oct	23.7	Und	–	1.3E+03	1.1E+03	Und	5.3E+02
	18 Oct	22.7	Und	–	1.1E+03	3.4E+03	1.9E+03	Und
	16 Nov	19.4	Und	–	Und	Und	Und	Und
	21 Dec	16	Und	–	Und	Und	Und	Und
2018	25 Jan	12.4	4.3E+02	–	Und	Und	Und	Und
	20 Feb	11.7	Und	–	Und	4.1E+02	Und	Und
	22 Mar	13.5	Und	–	1.4E+03	Und	Und	Und
	18 Apr	16.7	Und	–	Und	Und	Und	Und
	23 May	20.6	Und	–	Und	Und	Und	Und

a A4 and A5, net pens for red sea bream juveniles that were introduced in May 2017; B2 and B3, net pens for red sea bream broodstock; C, sampling point outside the fish farm; D, sampling point at a different fish farm to which the surviving fish (RSIV carriers) were transferred; –, no data; Und, under the detection limit (1.0E+02 copies/liter seawater).

In this study, koi herpesvirus (KHV), which has a similar morphology and particle size to RSIV, was supplemented to the seawater samples as the external standard virus between April 2018 and November 2019 (Table S1). The mean recovery rate of KHV in the eDNA was 60.2% ± 16.5% (±standard deviation). Assuming a similar recovery rate of RSIV, the quantitative results for RSIV in eDNA were considered to be the actual dynamics of RSIV in seawater.

### Nucleotide sequences derived from virus isolate, fish, and eDNA.

Nucleotide sequences of the MCP gene among virus isolate RS-17, juvenile fish, broodstock, and eDNA were compared to confirm whether the same virus origin was associated with the outbreaks in 2016 and 2017. The nucleotide sequence of the MCP gene for the RS-17 isolate (accession number LC605053) was identical to those of RSIV isolates such as RSIV-7 (accession number AB666334) from red sea bream and RSIV HyoDS-13 (accession number LC150886) from devil stinger (*Inimicus japonicus*), which have been reported in Japan (28, 45). Phylogenetic tree analysis indicated that the virus isolate was classified as RSIV genotype clade 2 (29, 30). The sequence analysis was performed for 17 samples derived from the eDNA, surviving fish, and broodstock (Table S2). Although the RSIV isolates in Japan can be divided into several subtypes in RSIV genotype clade 2 by the sequenced region (unpublished data), all the nucleotide sequences in analyzed samples were identical to that of the isolated virus RS-17 (data not shown).

## DISCUSSION

In the present study, viral dynamics in environmental seawater during an RSIVD epidemic in a fish farm were demonstrated following eDNA monitoring. Although several studies have focused on viral dynamics in environmental water (12–14, 46, 47), few studies have been undertaken to identify the source of infection in fish farms by monitoring eDNA. Using the iron flocculation method (15–17), the RSIV genome was detected in the eDNA at least 5 days earlier than the onset of mortality. This suggests that the method is useful for determining the status of disease progression for reared fish in tanks or net pens. This method can also be used to estimate the infection source by monitoring the viral dynamics of surviving fish or broodstock net pens. Furthermore, the fact that viral loads at sampling point C, which was approximately 100 m from the fish farm, were basically lower than those at sampling point A or B suggested that the viral loads in the seawater were highly associated with the reared fish near the sampling point. Viral loads in environmental water can be significantly affected by water currents and physical distance. Finite-volume ocean circulation and particle-tracking models have been applied to simulate the waterborne transmission of infectious hematopoietic necrosis virus (IHNV) among Atlantic salmon (*Salmo salar*) farms (48). Model simulations for virus dispersion in fish farms will be valuable in future estimations of transmission risks among net pens or fish farms.

Asymptomatically RSIV-infected broodstocks were regarded as the infection source for the RSIVD outbreak in the Japanese fish farm in 2017. Although the surviving fish were the most probable source of infection initially, they were not associated with the outbreak, as they were eliminated from the fish farm before the introduction of new juveniles. The high viral load of the broodstock net pens immediately before the rise in mortality levels strongly suggests that RSIV was shed from the broodstock and transmitted to the juveniles. The presence of identical nucleotide sequences among the seawater and fish tissue samples also supports this estimation.

At sampling point D, to which the surviving fish in 2016 were moved, no virus was detected 1 year after the relocation, even when the fish reached approximately 1 kg in 2018. Therefore, it is supposed that there may be a difference in the infection features between the RSIV-infected broodstock and the surviving fish. The RSIV-infected broodstock could represent a persistent infection that was distinct from that of the surviving fish. Since RSIVD has often been reported in juveniles, mature fish, such as the broodstock, may have acquired some resistance to RSIV infection (31, 49). As shown in this study (Table 2), however, the broodstock were indeed infected with RSIV without disease signs or mortality. Furthermore, the RSIV-infected broodstock seemed to shed the virus into the seawater for a prolonged period compared to the period of shedding of the surviving fish, and they propagated numerous virus particles in late June 2017. The persistent RSIV infections may have been activated due to weakened immune systems caused by spawning stresses, as the season seemed to be immediately after spawning for the broodstock that spontaneously matured in the net pens (50). In addition, an increase in water temperature, which is optimal for RSIV replication, could be another factor promoting virus activation (41). Furthermore, surviving fish that have experienced severe RSIVD could pose a risk as the infection source. During experimental infections in barred knifejaws, the RSIV genome was detected in surviving fish for up to 180 days (51). Thus, RSIV seems to persist in infected fish for a prolonged period, and hence, RSIV-infected surviving fish are often suspected to be an infection source for spontaneous outbreaks in fish farms (39, 40). However, the viral loads at sampling point D, where the surviving fish were reared, indicated that they shed few viruses into the environmental seawater, even though RSIV was still detected in the fish 10 months after the outbreak. This study suggests that even in cases where the viral genome is present in organs, there are differing risks of the surviving fish becoming sources of infection.

In the present study, disease progression of RSIVD in the fish farm, that is, an increase in daily mortality along with rising water temperature, was clearly synchronous with the viral dynamics in the environmental seawater. The optimal growth temperature of RSIV is approximately 25°C, determined by growth experiments using cell cultures (52, 53). There are several reports that the water temperature during RSIVD epidemics is more than 25°C in spontaneously occurring cases (19, 23, 54). When daily mortality exceeded 1,000 fish and 10,000 fish in the present study, the water temperature measured 26.8°C and 28.6°C, respectively. A previous study for field vaccine trials indicated that the water temperature during the mortality peak was 27°C (36). For RSIV-infected barred knifejaws, the period between infection and death was shortest when the infected fish were reared at 28 to 30°C (42, 51, 55), due to relatively more rapid replication of the virus at 28°C compared with its rate of replication at lower temperatures (55). A water temperature higher than 27°C seems to be the threshold that determines the magnitude of RSIVD outbreaks.

Adding an external standard virus to a water sample before iron flocculation is an effective method to estimate the recovery rate of the target virus. The recovery rate of RSIV by iron flocculation has been previously demonstrated to be approximately 80% in an experimental setting (16). However, we hypothesized that the recovery rate varied depending on the environmental water conditions during the monitoring period of 3 years. Thus, KHV was added into the seawater samples before iron flocculation as the external standard virus to estimate the RSIV recovery rates between April 2018 and November 2019. KHV particles resemble RSIV in terms of being enveloped virions and having a similar particle size and a double-stranded DNA (dsDNA) genome (56). Furthermore, KHV will not be detected in the seawater environment because the host range is limited to freshwater fish (56). Thus, KHV was effective as the external standard virus for RSIV detection. The recovery rate of KHV seemed to be stable throughout the 1 year of its use, indicating that the RSIV dynamics obtained in this study were most likely accurate. However, we could not perform the RSIV copy number correction based on the KHV recovery rate because we did not use the external standard virus when RSIVD occurred in 2017. Previous studies detecting KHV from environmental water used lambda phage as the external standard virus, and the KHV copy number in each sample was corrected according to its recovery rate (13, 14). A similar system should be established for more accurate monitoring of RSIV by eDNA in the future.

### Concluding remarks.

The results of the present study suggest that the risk of RSIV-infected fish as an infection source could differ between broodstock and surviving fish. Although they were indistinguishable based only on real-time PCR of tissue samples, the viral dynamics in the seawater showed that persistently infected broodstock had higher risks of being the infection source via virus shedding. Monitoring of eDNA using the iron flocculation method will be a valuable tool to appropriately understand infection sources and routes and will facilitate improved control of future RSIVD epidemics in fish farms. The results of the present study provide a tool based on eDNA that has potential application in aquaculture for the monitoring and control of fish diseases and could facilitate future research.

## MATERIALS AND METHODS

### Fish farm.

A fish farm located in the western part of Japan was investigated over 3 years between October 2016 and November 2019. The fish farm mainly reared juvenile and broodstock red sea bream in net pens, in addition to other fish species. The fish reared in the fish farm during the investigation are shown in Fig. 1A. RSIV-free red sea bream juveniles were produced annually using the fish farm broodstock. The juveniles were initially reared in land tanks and were moved to net pens after growing up. At the end of March 2017, before the outbreak of RSIVD, approximately 7,000 broodstocks were reared in the fish farm. The broodstocks had no record of RSIVD in the previous 4 years (2013 to 2016). The water temperature throughout the sampling period was measured daily using a thermometer set at an underwater depth of 1 m in the net pen. The daily mortality numbers during the outbreak in 2017 were obtained by manual counting by the fish farm staff. The mortality rate was calculated by the daily mortality and the total number of juveniles introduced in May 2017 (372,000 fish).

### Epidemiological information of RSIVD outbreak.

In the fish farm, approximately 247,000 and 573,000 red sea bream juveniles died due to RSIVD outbreaks in 2015 and 2016, respectively. The fish that survived RSIVD in 2016 (approximately 469,000 fish), which had the potential to be an infection source, were transferred to another bay area (sampling point D) by April 2017 to prevent horizontal transmission from the RSIV carriers to newly introduced juveniles. Red sea bream juveniles (372,000 fish) were then introduced into the fish farm in May 2017 (sampling point A). They were produced in a land tank and reared in another bay area to avoid horizontal transmission from the surviving fish in 2016. The newly introduced juveniles were confirmed to be RSIV negative through conventional PCR (32), which was performed by the fish farm (data not shown). They were not vaccinated for RSIVD. The first juvenile mortality due to RSIVD was recorded on 27 June 2017. The number of daily deaths was less than 1,000 fish until July 12, when daily mortality increased to more than 2,000 fish on July 13 and continued to increase with increasing water temperature (Fig. 2). On July 25, the number of daily deaths and water temperature reached 27,969 fish and 29.5°C, respectively, which represented the peak values for the outbreak in 2017. Mortality then gradually decreased and ceased by the end of August 2017. By April 2018, all fish surviving in 2017 were transferred to another bay area and approximately 90% of red sea bream broodstock were sold (Fig. 1). No red sea bream juveniles were introduced into the fish farm in 2018 and 2019. No RSIVD was recorded in the red sea bream broodstock or the other fish species reared on the fish farm between 2015 and 2019.

### eDNA samples from seawater.

Seawater was collected in 500-ml samples at a depth of 3 m using a water sampler (Yoshino Measurement K.K., Tokyo, Japan) more than once a month between October 2016 and November 2019 in the juvenile net pens (A1 to A5), the broodstock net pens (B1 to B3), and a point outside the fish farm (C) (Fig. 1B). The same method of seawater sampling was performed between June 2017 and June 2019 at another bay area (D), where fish surviving RSIVD in 2016 were transferred in April 2017. Heat-inactivated koi herpesvirus (KHV) NRIA0301 isolate (57) was added to the seawater samples between April 2018 and November 2019 as the external standard virus before the concentration process. Then, the seawater was concentrated using iron flocculation and eDNA was extracted, with minor modifications (16). Briefly, 50 μl of an FeCl_3_ solution (4.83 g FeCl_3_·6H_2_O in 100 ml distilled water) was added to 500 ml of the sample seawater. Subsequently, the iron-supplemented seawater (1 mg Fe liter^−1^) was gently stirred for 1 h using a magnetic stirrer. The seawater was filtered with an 0.8-μm-pore-size polycarbonate filter (Advantec Ltd., Tokyo, Japan) under reduced pressure to trap the resulting virus flocculate. The flocculate-trapped filter was transferred to a 1.5-ml microtube and stored at −80°C until eDNA extraction. The eDNA was directly extracted from the filter using a DNeasy blood and tissue kit (Qiagen K.K., Tokyo, Japan). Two hundred microliters of ATL solution and 20 μl of proteinase K supplied with the kit were added to the filter and incubated at 56°C for 2 h. After applying a further 200 μl of AL solution to the filter, it was incubated at 56°C for over 30 min. The lysate was mixed with 200 μl ethanol, and DNA purification was performed according to the manual’s directions. The final DNA elution was performed with 0.2 ml of the elution buffer supplied with the kit. This resulted in a 2,500-times concentrated eDNA from the 500-ml sample of seawater.

### Fish samples.

Red sea bream associated with the RSIVD outbreaks were investigated in 2016 and 2017 (Fig. 1A). Fish surviving after the RSIVD outbreak in August 2016 were sampled on 7 November (5 fish) in 2016 and on 10 January (15 fish), 12 April (15 fish), and 8 June (10 fish) in 2017. For the samples derived from the RSIVD outbreak in July 2017, five dead fish were sampled on 27 July, and five surviving fish were each sampled on 21 September and 6 October 2017. Each of the caudal fins, gills, intestines, livers, kidneys, and spleens of the surviving fish sampled on 7 November 2016 and the spleens of the other fish samples were subjected to DNA extraction. With respect to the broodstock sampling, three 4-year-old red sea breams (1.21 ± 0.15 kg) were sampled from a net pen with no record of RSIVD outbreaks on October 18, 2017. The broodstock was produced in 2013 and reared on the same fish farm until 2017. Before DNA extraction, approximately 1 g of each tissue was collected from the brain, caudal fin, gills, gonad, stomach, pyloric caeca, intestine, liver, kidney, and spleen. The tissue was minced with scissors and mixed well for the homogenization of RSIV in the tissue. Total DNA was extracted from approximately 30 mg of the tissue sample using a Maxwell 16 system DNA purification kit (Promega Corporation, Madison, WI, USA). The remaining tissues that were not used for DNA extraction were stored at −80°C for virus isolation as described below.

### Real-time PCR.

The DNA samples derived from the seawater and fish samples were analyzed by real-time PCR targeting the MCP gene of RSIV (58). To evaluate the recovery rate of KHV as the external standard virus in the seawater sample, another real-time PCR (59) was performed separately. The primer/probe set for RSIV consisted of RSIV-MCP186F (5′-CGG CCA GGA GTT TAG TGT GAC T-3′), RSIV-MCP288R (5′-GCT GTT CTC CTT GCT GGA CG-3′), and hydrolysis probe RSIV-MCP239P (5′-FAM-TGT GGC TGC GTG TTA AGA TCC CCT CCA-BHQ1-3′). The primer/probe set for KHV consisted of KHV-86f (5′-GAC GCC GGA GAC CTT GTG-3′), KHV-163r (5′-CGG GTT CTT ATT TTT GTC CTT GTT-3′), and hydrolysis probe KHV-109p (5′-FAM-CTT CCT CTG CTC GGC GAG CAC G-TAMRA-3′). Each well contained 20 μl of reaction mixture consisting of 2 μl nucleic acid template and 10 μl of FastStart essential DNA probes master mix (Roche Diagnostics K.K., Tokyo, Japan), with a final concentration of 200 nM for each primer and probe. For the positive control, a serial 10-fold dilution of the plasmid containing the MCP region of RSIV KagYT-96 isolate (58) was used to draw the standard curve. Real-time PCR assays were performed using a LightCycler 96 Instrument (Roche Diagnostics). The amplification thermal profile consisted of one cycle of 95°C for 10 min and 45 cycles of denaturation and annealing at 95°C for 10 s and 60°C for 30 s. Samples were tested in duplicate. The eDNA was determined to be positive when the quantification cycle (*C_q_*) value was less than 40 and when its amplification curve was similar to that of the positive control (Fig. S1). Furthermore, some real-time PCR amplicons (*C_q_* value of 35 to 36) of the eDNA samples were confirmed to be from the RSIV genome by sequencing analysis, and hence, the assay had high specificity even in the eDNA samples (Fig. S1). On the other hand, we noticed real-time PCR inhibition, because the RSIV copy number was lower than the predicted value even during the RSIVD outbreak in 2017. Then, all the eDNA samples until April 2018 were serially 2-fold diluted (1/2 to 1/8) and subjected to real-time PCR before the external standard virus was added. The lowest dilution of eDNA from which RSIV was stably detected was selected for the result after it was normalized by the dilution factor (1/2 or 1/4 dilution was often used). The inhibition was confirmed between June and September in 2017. The recovery rate of KHV was calculated using the copy number derived from the eDNA samples and KHV DNA extracted from the virus solution which was added to the seawater. The real-time PCR inhibition was monitored by the KHV recovery rate. Since the inhibition was confirmed in July 2018, the diluted eDNA was reanalyzed as described above.

### Virus isolation.

The fish samples (spleen) from which the RSIV genome was detected by real-time PCR were subjected to virus isolation using the SKF-9 cell line that originated from larvae of spotted knifejaw *Oplegnathus punctatus* (58). The cell line was maintained at 25°C with Hanks’ minimum essential medium (HMEM; GIBCO/Thermo Fisher Scientific K.K., Tokyo, Japan) supplemented with 10% fetal bovine serum (FBS) and 1× antibiotic-antimycotic (Gibco/Thermo Fisher Scientific). The spleen was homogenized with 10 times the volume of medium (HMEM with 10% FBS and 1× antibiotic-antimycotic). The supernatant of the homogenate was clarified by centrifugation at 800 × *g* for 10 min at 4°C and passed through a 0.45-μm membrane filter. A 50-μl sample of the filtrate was inoculated onto SKF-9 cells, which were seeded in 25-cm^2^ cell culture flasks (Corning Life Sciences, Inc., Corning, NY, USA) or 24-well plates (Thermo Fisher Scientific) the day before sample inoculation. The inoculated cells were incubated at 25°C for 14 days. The cell culture supernatant was passaged into fresh SKF-9 cells. Since cytopathic effects (CPE) were only observed in the samples from the dead fish from 27 July 2017, the passaged supernatant was stored at −80°C as RSIV isolate RS-17.

### Sequencing analysis.

The major capsid protein (MCP) gene of RSIV derived from the seawater, fish, and isolated virus samples was determined (Table S2). PCR primers for the amplification of the total length of the MCP gene consisted of RSIV MCP-F0 (5′-GTC GGA CTG TTG GTC TTG CT-3′) and RSIV MCP-R0 (5′-CAG CAT GCC TTG CAT ACG-3′). PCR amplification was performed using KOD FX NEO (Toyobo Co. Ltd., Osaka, Japan) or TaKaRa *Ex Taq* hot-start version (TaKaRa Bio, Inc., Shiga, Japan). The amplification thermal profile consisted of one denaturation cycle of 94°C for 2 min, followed by 35 cycles of denaturation at 94°C for 30 s, annealing at 58°C or 60°C for 30 s, and extension at 72°C for 90 s, followed by a final extension step at 72°C for 2 min. A nested PCR was performed for the samples derived from the seawater and fish, which had low viral loads. The amplicon from the above-described PCR, which was diluted 100 times with distilled water, was used as the template. The PCR primers for the nested PCR consisted of RSIV MCP-F1 (5′-GAA AAA CGA GGC CGA TCA TA-3′) and RSIV MCP-R1 (5′-GCA TAC GCT ATG GCC ACA AT-3′). The amplification thermal profile consisted of one denaturation cycle of 94°C for 2 min, followed by 30 or 35 cycles of denaturation at 94°C for 30 s, annealing at 60°C for 30 s, and extension at 72°C for 90 s, followed by a final extension step at 72°C for 2 min. The amplicons of 1,524 bp (RSIV MCP-F0/R0) and 1,475 bp (RSIV MCP-F1/R1) were purified using Agencourt AMPure XP beads (Beckman Coulter, Inc., Brea, CA, USA) and directly sequenced by a DNA sequencing service (Fasmac Co. Ltd., Kanagawa, Japan).

### Data availability.

The nucleotide sequence of the MCP gene for the virus isolate, RS-17, was submitted to the DNA Data Bank of Japan (DDBJ) under accession number LC605053.
