# Supplementary material for: Application of Environmental DNA for Monitoring Red Sea Bream Iridovirus at a Fish Farm
Source: Microbiol Spectr. 2021 Oct 27;9(2):e00796-21. doi: 10.1128/Spectrum.00796-21 (PMC8549737; doi:10.1128/Spectrum.00796-21)
Supplement: SUPPLEMENTAL FILE 1 — Supplemental material. Download Spectrum.00796-21-s0001.pdf, PDF file, 0.3 MB [file spectrum.00796-21-s0001.pdf]

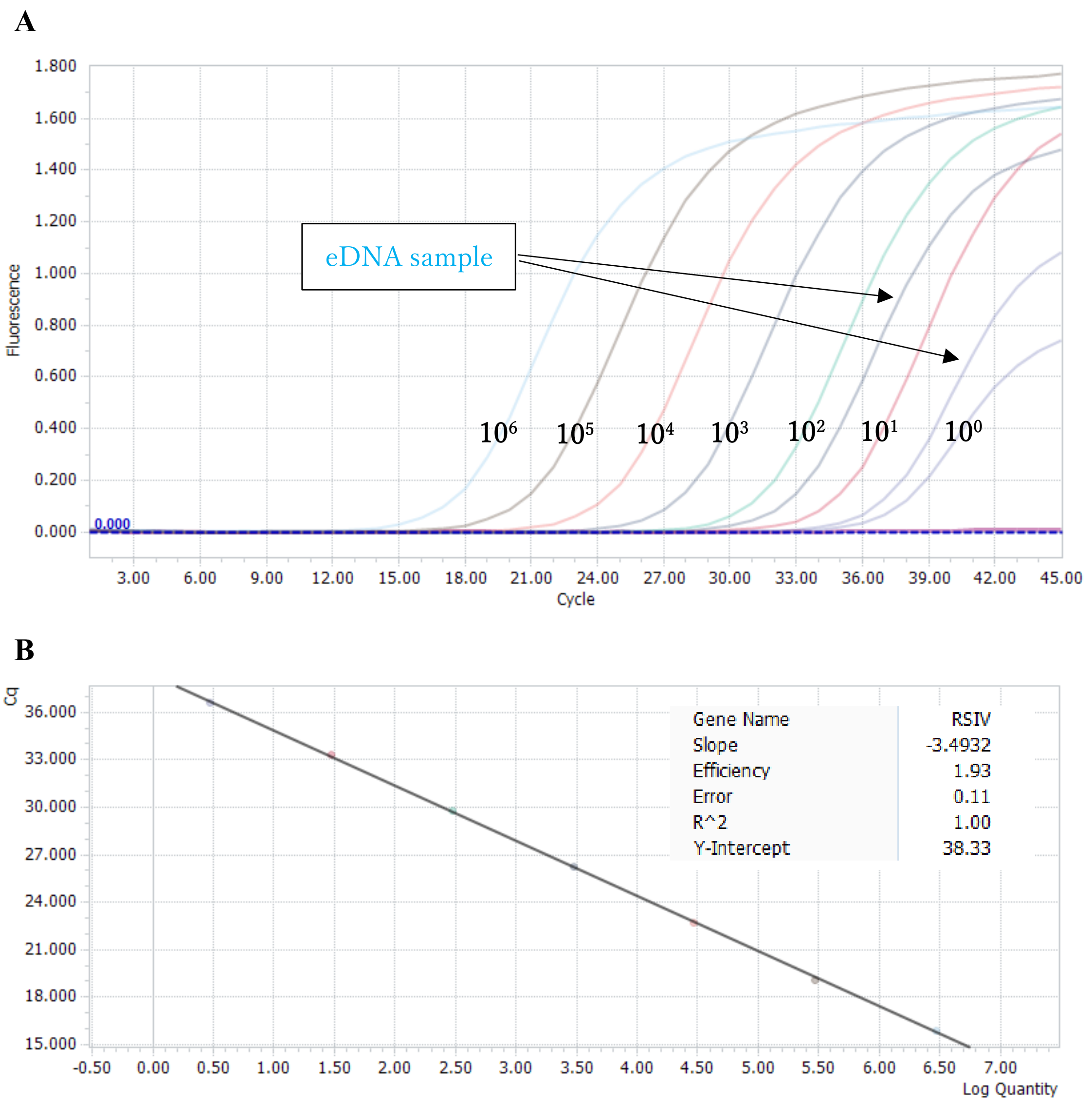

Fig. S1. Amplification curves (A) and standard curve (B) of the real-time PCR used in this study. Amplification curves consist of eDNA samples and a serially diluted plasmid in which the RSIV MCP gene was incorporated. The amplification curve derived from the eDNA samples were confirmed to be the RSIV genome by the amplicon sequencing after the real-time PCR reaction.

Table S1. Viral load in the eDNA samples.

Year	Date	Water temperature (°C)	RSIV genome number (copies/L seawater) at the sampling point[*1]										Recovery rate of KHV (external control virus) at the sampling point[*2]									
			A1	A2	A3	A4	A5	B1	B2	B3	C	D	A1	A2	A3	A4	A5	B1	B2	B3	C	D
2016	10/18	24.0	1.5E+02	1.5E+02	5.6E+02	-[*3]	-	4.2E+03	-	-	-	-	-	-	-	-	-	-	-	-	-	-
	10/25	23.0	4.7E+02	6.6E+02	2.7E+02	-	-	1.6E+04	-	-	Und	Und	-	-	-	-	-	-	-	-	-	-
	11/7	21.0	2.6E+02	Und	Und	-	-	Und	-	-	Und	Und	-	-	-	-	-	-	-	-	-	-
2017	1/10	14.4	Und[*4]	Und	Und	-	-	Und	-	-	Und	Und	-	-	-	-	-	-	-	-	-	-
	2/9	12.5	Und	Und	Und	-	-	Und	-	-	Und	Und	-	-	-	-	-	-	-	-	-	-
	3/21	12.7	Und	Und	Und	-	-	Und	-	-	Und	Und	-	-	-	-	-	-	-	-	-	-
	4/12	15.3	Und	Und	Und	-	-	Und	-	-	Und	Und	-	-	-	-	-	-	-	-	-	-
	5/17	19.4	-	-	-	Und	-	-	Und	Und	Und	-	-	-	-	-	-	-	-	-	-	-
	6/8	21.0	-	-	-	Und	-	-	Und	Und	Und	1.9E+03	-	-	-	-	-	-	-	-	-	-
	6/22	22.6	-	-	-	8.3E+03	1.5E+04	-	1.6E+05	8.0E+04	Und	-	-	-	-	-	-	-	-	-	-	-
	7/6	26.5	-	-	-	4.6E+03	2.0E+04	-	8.7E+02	2.3E+03	5.0E+02	Und	-	-	-	-	-	-	-	-	-	-
	7/13	26.8	-	-	-	2.8E+05	5.6E+05	-	1.6E+03	2.0E+03	1.6E+03	Und	-	-	-	-	-	-	-	-	-	-
	7/20	28.8	-	-	-	6.3E+05	8.5E+05	-	2.1E+03	4.5E+03	2.8E+03	3.8E+02	-	-	-	-	-	-	-	-	-	-
	7/27	27.3	-	-	-	3.8E+06	3.0E+06	-	1.8E+04	2.6E+03	1.1E+04	Und	-	-	-	-	-	-	-	-	-	-
	8/3	26.6	-	-	-	6.5E+03	4.8E+02	-	6.6E+03	5.6E+03	1.2E+04	Und	-	-	-	-	-	-	-	-	-	-
	8/9	27.6	-	-	-	1.0E+04	-	-	1.7E+03	4.9E+03	Und	8.0E+02	-	-	-	-	-	-	-	-	-	-
	8/17	27.2	-	-	-	1.2E+03	-	-	Und	Und	Und	Und	-	-	-	-	-	-	-	-	-	-
	8/24	28.7	-	-	-	2.2E+03	-	-	4.2E+03	9.0E+03	Und	Und	-	-	-	-	-	-	-	-	-	-
	9/7	25.9	-	-	-	4.9E+03	-	-	2.5E+03	2.4E+03	1.9E+03	Und	-	-	-	-	-	-	-	-	-	-
	9/21	24.8	-	-	-	Und	-	-	Und	Und	6.9E+02	Und	-	-	-	-	-	-	-	-	-	-
	10/6	23.7	-	-	-	Und	-	-	1.3E+03	1.1E+03	Und	5.3E+02	-	-	-	-	-	-	-	-	-	-
	10/18	22.7	-	-	-	Und	-	-	1.1E+03	3.4E+03	1.9E+03	Und	-	-	-	-	-	-	-	-	-	-
	11/16	19.4	-	-	-	Und	-	-	Und	Und	Und	Und	-	-	-	-	-	-	-	-	-	-
	12/21	16.0	-	-	-	Und	-	-	Und	Und	Und	Und	-	-	-	-	-	-	-	-	-	-
2018	1/25	12.4	-	-	-	4.3E+02	-	-	Und	Und	Und	Und	-	-	-	-	-	-	-	-	-	-
	2/20	11.7	-	-	-	Und	-	-	Und	4.1E+02	Und	Und	-	-	-	-	-	-	-	-	-	-
	3/22	13.5	-	-	-	Und	-	-	1.4E+03	Und	Und	Und	-	-	-	-	-	-	-	-	-	-
	4/18	16.7	-	-	-	Und	-	-	Und	Und	Und	Und	-	-	-	57.5%	-	-	60.6%	69.4%	74.0%	57.5%
	5/23	20.6	-	-	-	Und	-	-	Und	Und	Und	Und	-	-	-	55.1%	-	-	65.2%	61.4%	55.8%	52.5%
	6/28	23.8	-	-	-	Und	-	-	Und	Und	Und	Und	-	-	-	47.7%	-	-	48.2%	48.6%	45.7%	56.3%
	7/26	29.0	-	-	-	Und	-	-	Und	Und	Und	Und	-	-	-	55.5%	-	-	63.2%	38.7%	65.8%	3.7%
	8/23	28.0	-	-	-	Und	-	-	Und	Und	Und	Und	-	-	-	60.0%	-	-	60.6%	50.3%	58.1%	46.5%
	9/28	24.8	-	-	-	Und	-	-	Und	Und	Und	Und	-	-	-	55.8%	-	-	47.3%	63.5%	64.1%	51.5%
	10/29	20.6	-	-	-	Und	-	-	Und	Und	Und	Und	-	-	-	49.2%	-	-	48.4%	39.3%	41.5%	41.9%
	11/22	18.0	-	-	-	Und	-	-	Und	Und	Und	Und	-	-	-	49.3%	-	-	62.0%	54.1%	56.1%	61.9%
	12/27	17.0	-	-	-	Und	-	-	Und	Und	Und	Und	-	-	-	92.4%	-	-	78.7%	83.4%	85.9%	67.9%
2019	1/28	14.8	-	-	-	Und	-	-	Und	Und	Und	Und	-	-	-	49.6%	-	-	77.6%	80.3%	81.7%	60.6%
	2/26	13.8	-	-	-	Und	-	-	Und	Und	Und	Und	-	-	-	38.0%	-	-	64.5%	57.3%	56.0%	49.7%
	3/30	15.8	-	-	-	Und	-	-	Und	Und	Und	Und	-	-	-	83.7%	-	-	81.9%	77.0%	78.8%	83.7%
	4/21	17.2	-	-	-	Und	-	-	Und	Und	Und	Und	-	-	-	58.1%	-	-	76.5%	72.7%	66.9%	81.3%
	5/27	21.8	-	-	-	Und	-	-	Und	Und	Und	Und	-	-	-	44.8%	-	-	53.4%	52.9%	29.4%	53.1%
	6/28	28.8	-	-	-	Und	-	-	Und	Und	Und	Und	-	-	-	64.0%	-	-	53.1%	61.9%	54.9%	66.1%
	7/23	25.4	-	-	-	Und	-	-	3.8E+02	Und	Und	-	-	-	-	64.0%	-	-	34.1%	57.3%	42.9%	-
	8/27	26.7	-	-	-	Und	-	-	Und	Und	Und	-	-	-	-	71.4%	-	-	89.3%	70.6%	109.7%	-
	9/30	25.2	-	-	-	Und	-	-	Und	Und	Und	-	-	-	-	90.6%	-	-	68.3%	74.1%	49.9%	-
	10/30	21.0	-	-	-	Und	-	-	2.7E+02	Und	Und	-	-	-	-	63.9%	-	-	75.4%	79.4%	72.6%	-
	11/26	19.0	-	-	-	4.0E+02	-	-	Und	Und	Und	-	-	-	-	29.5%	-	-	51.6%	42.4%	19.1%	-

[*1]: A1-A5 represent the net-pens for the red sea bream juveniles. B1-B3 represent the net-pens for the red sea bream broodstock. C is the area outside of the fish farm. D is athe net-pen at the other fish farm where the surviving fish (RSIV carrier) were transferred.

[*2]: The external control virus was added between April 2018 and November 2019.

[*3]: No data

[*4]: Under the detection limit (1.0E+02 copies/L seawater)

Table S2. Sample information for the sequencing analysis of the MCP gene of RSIV.

No.	Sample name	Sampling date	Sample information
1	RS-17	2017/7/27	Virus isolate from dead fish
2	161018-A2	2016/10/18	Seawater from Juvenile net-pen (A2)
3	161018-A3	2016/10/18	Seawater from Juvenile net-pen (A3)
4	161018-B1	2016/10/18	Seawater from Broodstock net-pen (B1)
5	161025-B1	2016/10/25	Seawater from Broodstock net-pen (B1)
6	170622-B2	2017/6/22	Seawater from Broodstock net-pen (B2)
7	170622-B3	2017/6/22	Seawater from Broodstock net-pen (B3)
8	170706-B3	2017/7/6	Seawater from Broodstock net-pen (B3)
9	170706-A4	2017/7/6	Seawater from Juvenile net-pen (A4)
10	170706-A5	2017/7/6	Seawater from Juvenile net-pen (A5)
11	170713-A4	2017/7/13	Seawater from Juvenile net-pen (A4)
12	170713-A5	2017/7/13	Seawater from Juvenile net-pen (A5)
13	170110-surviving 1	2017/1/10	Spleen of surviving fish
14	170110-surviving 2	2017/1/10	Spleen of surviving fish
15	170728-dead1	2017/7/27	Spleen of dead fish
16	170728-dead2	2017/7/27	Spleen of dead fish
17	171018-BS1	2017/10/18	Spleen of broodstock
18	171018-BS2	2017/10/18	Spleen of broodstock
